# Taxonomic and Phylogenetic Reassessment of *Pyrgidium* (*Mycocaliciales*) and Investigation of Ascospore Morphology

**DOI:** 10.3390/jof8090966

**Published:** 2022-09-15

**Authors:** Vinodhini Thiyagaraja, Damien Ertz, Robert Lücking, Dhanushka N. Wanasinghe, André Aptroot, Marcela Eugenia da Silva Cáceres, Kevin D. Hyde, Wanaporn Tapingkae, Ratchadawan Cheewangkoon

**Affiliations:** 1 Department of Entomology and Plant Pathology, Faculty of Agriculture, Chiang Mai University, Chiang Mai 50200, Thailand; 2 Centre of Excellence in Fungal Research, Mae Fah Luang University, Chiang Rai 57100, Thailand; 3 Centre for Mountain Futures (CMF), CAS Key Laboratory for Plant Biodiversity and Biogeography of East Asia (KLPB), Kunming Institute of Botany, Chinese Academy of Sciences, Kunming 650201, China; 4 CIFOR-ICRAF China Program, World Agroforestry Centre, East and Central Asia, 132 Lanhei Road, Kunming 650201, China; 5 Research Department, Meise Botanic Garden, Nieuwelaan 38, BE-1860 Meise, Belgium; 6 Fédération Wallonie-Bruxelles, Service Général de l’Enseignement Supérieur et de la Recherche Scientifique, Rue A. Lavallée 1, BE-1080 Bruxelles, Belgium; 7 Botanischer Garten und Botanisches Museum, Freie Universität Berlin, Königin-Luise-Str. 6–8, 14195 Berlin, Germany; 8 Instituto de Biociências, Universidade Federal de Mato Grosso do Sul, Avenida Costa e Silva, s/n Bairro Universitário, Campo Grande CEP 79070-900, Brazil; 9 Departamento de Biociências, Universidade Federal de Sergipe, Itabaiana CEP 49500-000, Brazil; 10 Department of Animal and Aquatic Sciences, Faculty of Agriculture, Chiang Mai University, Chiang Mai 50200, Thailand

**Keywords:** *Ascomycota*, morphology, *Mycocaliciaceae*, PCA, saprotrophs, SEM

## Abstract

*Mycocaliciales* comprise non-lichenized either saprotrophic or lichenicolous fungi which occur in temperate and tropical regions. The mazaediate, saprotrophic and monospecific genus, *Pyrgidium*, is currently assigned to this order, yet the phylogenetic placement of the genus has remained uncertain due to the absence of molecular data. In order to investigate the systematic position of *Pyrgidium*, two specimens collected in Brazil and Thailand, respectively, were used to generate mtSSU, SSU, LSU and ITS sequences. However, given that most other representatives of this order only have LSU and ITS sequences available, the phylogenetic reconstruction was limited to these two markers. The phylogenetic analyses confirmed placement of the genus within *Mycocaliciales*, the genus possessing a sister group relationship with the lichenicolous genus *Sphinctrina*. Detailed morphological descriptions and illustrations are provided, including those for type specimens of the various synonyms subsumed under the hitherto only accepted species, *Pyrgidium montellicum* (Beltr.) Tibell. The ascospore morphology was investigated using compound and scanning electronic microscopy (SEM). Principal component analysis (PCA) was performed for the ascospore size using PC-ORD 7. The molecular data and re-examination of the type specimens support the monospecific nature of this genus.

## 1. Introduction

Calicioid or mazaediate fungi are characterized by the production of ascospore masses accumulating on top of the ascomata after the disintegration of the asci [1,2]. Mazaediate fungi represent a heterogenous group of lichenized and non-lichenized lineages, traditionally assigned to the largely lichenized order *Caliciales* [3,4,5,6,7]. Vainio [8] pointed out the variable nutritional mode of the genera in this group and suggested excluding the non-lichenized genera from *Caliciales*, highlighting the absence of a photobiont when establishing the genus *Mycocalicium*. Schmidt [9] introduced the family *Mycocaliciaceae* to accommodate the non-lichenized calicioid genera, including *Mycocalicium*, *Chaenothecopsis, Phaeocalicium, Stenocybe* and *Strongyleuma*. Nevertheless, *Caliciaceae*, *Mycocaliciaceae* and *Sphinctrinaceae* remained in the core group of *Caliciales*, based on their shared morphological characteristics, such as stalked ascomata, dark, sclerotized hyphae and melanized ascospores [5,6,10,11]. Notably*, Mycocaliciaceae* have also independently evolved to have the trait of active spore dispersal without producing mazaedia [10].

Tibell [5,12] emphasized the heterogenous nature of *Caliciales*, and this was eventually resolved through phylogenetic analyses, which led to the placement of the calicioid lineages into different classes of *Ascomycota*, including *Arthoniomycetes*, *Eurotiomycetes*, *Lecanoromycetes* and *Leotiomycetes*, and within two subclasses and several orders of *Lecanoromycetes* [1,4,13]. Wedin and Tibell [4] showed that *Mycocaliciaceae* and *Sphinctrinaceae* form a monophyletic group within *Eurotiales*, whereas *Caliciaceae* are clustered close to *Lecanorales*. These placements were further supported by the nutritional biology and spore ornamentation [4]. Tibell and Wedin [6] then introduced *Mycocaliciales* to accommodate *Mycocaliciaceae* and *Sphinctrinaceae* in the *Eurotiomycetes*, supporting findings of other studies [4,5,6,7,14]. Hibbett et al. [15] established the subclass *Mycocaliciomycetidae* for the single order *Mycocaliciales*, and this classification was accepted in further works [16,17,18,19,20,21,22,23,24]. *Mycocaliciaceae* and *Sphinctrinaceae* share morphological characteristics, such as sessile to stalked ascomata, a sclerotized, blackish brown exciple, cylindrical asci, and dark brown ascospores with smooth or ornamented walls [6]. 

The *Mycocaliciaceae* family encompasses algicolous, lichenicolous and lignicolous species on bark, plant exudates, wood and lichens [25,26,27,28,29]. *Sphinctrinaceae* was introduced by Choisy [30] as a monogeneric family to accommodate *Sphinctrina*, and later, Tibell [12] added *Pyrgidium* to this family. Species of *Sphinctrina* are exclusively lichenicolous [5,31], while *Pyrgidium* includes one (presumably saprobic) bark-inhabiting fungus [5]. Given their shared morphological features and lack of clear phylogenetic separation, the *Sphinctrinaceae* family was treated as a synonym of *Mycocaliciaceae* by Jaklitsch et al. [32], and this classification was followed in subsequent works [33,34]. 

The genus *Pyrgidium* was originally introduced by Nylander [35], with the type *P. bengaliense*. Nádvorník [36] combined *Trachylia leptoconia* Nyl. with *Pyrgidium*, while Tibell [37] transferred *Acolium montellicum* Beltr. to this genus. Tibell [5] considered *Pyrgidium* a monospecific genus, synonymizing *P. bengaliense* and *P. leptoconia* with *P. montellicum*. However, the genus was not studied in more detail afterwards, and the total number of species was defined as between one and three depending on the sources, including fungal databases and *Ascomycota* outlines [23,38,39]. The phylogenetic placement of *Pyrgidium* remained unresolved due to a lack of molecular data [6]. 

This study’s objective was to resolve the phylogenetic placement of *Pyrgidium* based on molecular analyses for the first time using LSU and ITS markers with the methods of maximum likelihood (ML) and Bayesian inference (BI) and, additionally, SSU and mtSSU to compare the sequence variation in materials from different tropical areas. Using both molecular and morphological data, we addressed the question of how many species can potentially be distinguished in this genus. Detailed morphological descriptions and illustrations are provided for both the freshly collected specimens and for the type specimens with the names previously assigned to *Pyrgidium.* The ascospore morphology of *Pyrgidium* was assessed with the aid of compound and SEM photographs, and PCA was performed to test the potential of specimens based on their ascospore sizes. We conclude that, at present, only one pantropical species, *P. montellicum*, should be recognized, agreeing with the previous findings of Tibell [5].

## 2. Materials and Methods

### 2.1. Sample Collection, Herbarium Examination and Morphological Studies 

Fresh material was collected in Brazil and Thailand to ensure a broad geographic representation of this taxon. Type specimens of *Pyrgidium* *bengaliense, Trachylia leptoconia*, and *Acolium montellicum* were borrowed from the Uppsala University herbarium (UPS). Macro-morphological structures were observed with a dissecting microscope (MOTIC SMZ-168) and photographed with a ZEISS Discovery v8 stereomicroscope with an AxioCam ERc 5s camera (Carl Zeiss, Jena, Germany). Hand sections of the ascomata were mounted and examined in water and 5% KOH, and micro-morphological features were examined using a NIKON Eclipse 80i (Nikon Corporation, Tokyo, Japan) compound microscope fitted with a CANON 750D digital camera. For the scanning electron microscopy, ascospores from fresh and herbarium specimens of *Pyrgidium* were placed on a carbon-covered SEM mount, sputtered with palladium and examined under a scanning electron microscope (AI-FE-SEM/T) with 5 KV energy. All microscopic measurements were performed with Tarosoft Image Frame Work (0.9.0.7), and images of the photoplates were processed with Adobe Photoshop CS6 Extended 10.0 (Adobe Systems, San Jose, CA, USA). The freshly collected specimens were deposited in the ISE herbarium (Federal University of Sergipe, Brazil) and in the MFLU herbarium (Mae Fah Luang University, Chiang Rai, Thailand). Faces of the fungi numbers were registered following Jayasiri et al. [40].

### 2.2. DNA Extraction, PCR Amplification and Sequencing

The DNA isolation was carried out using hand-made sections of ascomata by the direct PCR method, using an E.Z.N.A.^®^ Forensic DAT (D3591—01, Omega Bio-Tek, Norcross, GA, USA) DNA extraction kit and following the manufacturer’s instructions. DNA samples that were intended for use as a template for the PCR were stored at 4 °C to enable their use in regular work and duplicated at −20 °C for long-term storage. PCR was performed using specifications for each marker (Table 1). The purification and sequencing of the PCR products were performed by Tsingke Biotechnology Co., Ltd. (Kunming, China). The phylogenetic analyses were conducted following the recent protocol [41].

### 2.3. Phylogenetic Analyses

BLAST searches (NCBI) (https://www.ncbi.nlm.nih.gov; accessed on 15 January 2022) were performed for the newly generated sequences and, after the confirmation of their identity, the sequences were assembled in SeqMan [47] and deposited in GenBank (Table 2). For the phylogenetic analysis, we selected representative sequences of *Sphinctrinaceae* and *Mycocaliciaceae*, and for the outgroup taxa, we followed Tibell and Vinuesa [48]. The final combined LSU–ITS data set comprised 32 terminals, including five new sequences (Table 2). Given that most *Mycocaliciales* are only represented by LSU and ITS sequences, we did not include the newly generated SSU and mtSSU sequences in the analysis but assessed and deposited them separately: MFLU 21-0135; SSU (ON979668), Cáceres and Aptroot 11449; mtSSU (ON979677). We followed Dissanayake et al. [41] for the phylogenetic analyses. Multiple alignments of the LSU and ITS were first performed separately with MAFFT 7 (http://mafft.cbrc.jp/alignment/server), using the default settings [49]. Ambiguous regions and introns were manually adjusted or trimmed, where necessary, using BioEdit 7 [50]. The phylogenetic web tool “ALTER” [51] was used to convert the sequence alignments into the formats required for the ML and Bayesian analyses. The ML tree was generated using RAxML-HPC2 8.2.8 on XSEDE [52] on the CIPRES Science Gateway platform [53], with 1000 bootstrap pseudoreplicates. MrBayes 3.1.2 was used to perform the Bayesian analysis [54]. We employed MrModeltest 2.3 [55] to select the best-fitting model using the Akaike information criterion (AIC), and GTR + I + G was selected as the best-fitting model for each marker. Markov Chain Monte Carlo sampling (MCMC) was run for 5,000,000 generations, and the trees were sampled every 100th generation. The first 10% of the trees that represented the burn-in phase were discarded, and the remaining 90% were used to calculate the posterior probabilities (PP) for the majority rule consensus tree. The resulting trees were visualized in FigTree 1.4.0 [56] and subsequently edited in Microsoft PowerPoint (2013) and Adobe Photoshop CS6 version 10.0.

### 2.4. PCA

The PCA was performed in PC-ORD 7 to assess the size variation in the ascospores of *Pyrgidium*. The ascospore length and width, as well as the Q value (length:width ratio), were used as variables for the eight specimens of *P. montellicum* from various geographic regions, including Cáceres and Aptroot 11449, Kurz 1866, L-008798, L-996762, Lindig 2865, MFLU 21-0135, Tibell 8232 and Tibell 8306. Measurements were taken from 50 ascospores of each specimen. 

To test for significant differences in the ascospore size according to the geographic region by means of ANOVA with post hoc Tukey HSD, we grouped the measurements into three categories: (1) the Neotropics (Costa Rica, Colombia, Brazil), (2) Europe (Italy), and Paleotropics (India, Thailand). The ANOVA and the post hoc Tukey HSD were performed online (https://www.socscistatistics.com/tests/anova/default2.aspx, accessed on 15 January 2022).

## 3. Results

### 3.1. Phylogenetic Analyses 

**Figure 1:**
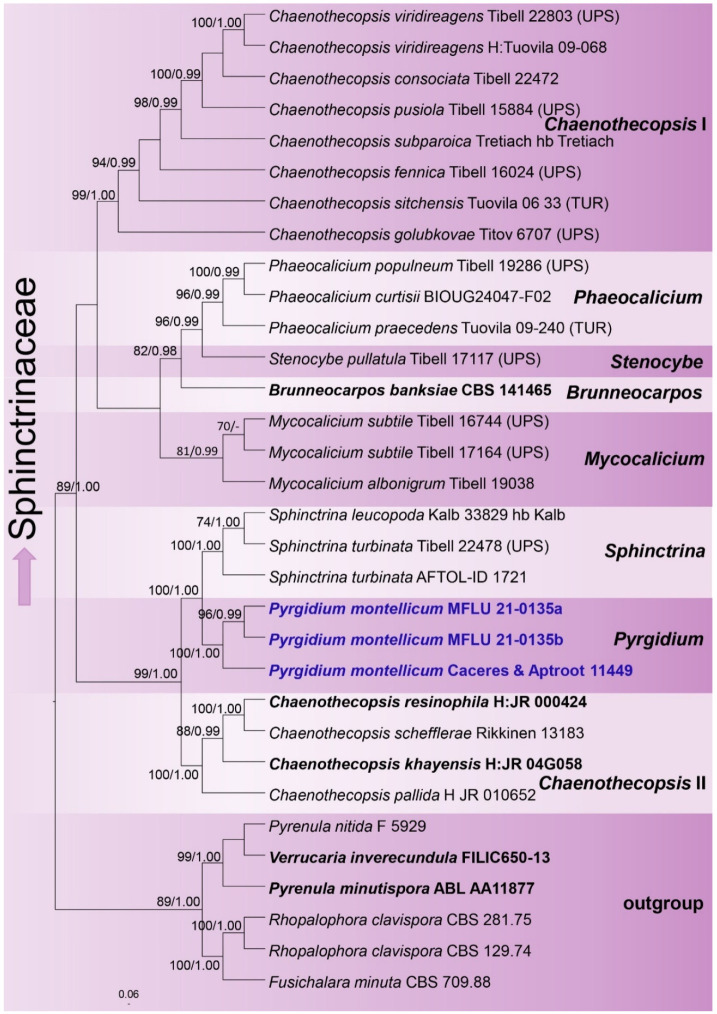
Best-scoring ML tree based on the analysis of the combined LSU and ITS sequence data. Bootstrap support values equal to or greater than 70% and Bayesian posterior probabilities (BP) equal to or greater than 0.95 are given as ML/BP above the branches next to the nodes. Ex-type strains of genera other than *Pyrgidium* are displayed in bold, and the new sequences generated in this study are indicated in blue. The tree was rooted with *Fusichalara minuta* (CBS 709.88), *Pyrenula minutispora* (ABL AA11877), *P. nitida* (F 5929), *Rhopalophora clavispora* (CBS 129.74), *R. clavispora* (CBS 281.75) and *Verrucaria inverecundula* (FILIC650-13), following Tibell and Vinuesa [48].

The final LSU–ITS dataset comprised 32 taxa with 1532 aligned characters, including gaps (LSU: 894; ITS: 638). The best-scoring ML tree was selected to represent the relationships between the taxa, with the final ML optimization likelihood value of –10603.813268 (Figure 1). The parameters for the GTR + I + G model of the combined LSU and ITS data were as follows: the estimated base frequencies A = 0.240252, C = 0.243881, G = 0.287821, T = 0.228046, and the substitution rates AC = 1.442589, AG = 2.659004, AT = 1.892966, CG = 1.070779, CT = 7.537779 and GT = 1.000000. Bayesian posterior probabilities from the MCMC were evaluated with the final average standard deviation of split frequencies = 0.001450. The topologies of the ML and the Bayesian tree were manually compared and were largely congruent.

The genera of *Mycocaliciales* were resolved as monophyletic clades, except for *Chaenothecopsis,* which appears to be polyphyletic. The genus *Pyrgidium* formed a sister clade with *Sphinctrina*, and both clades were strongly supported. *Pyrgidium* itself formed two clades, one with a single specimen from Brazil and the other with two sequences from Thailand, the latter two clustering with a high level of statistical support.

### 3.2. PCA

**Figure 2:**
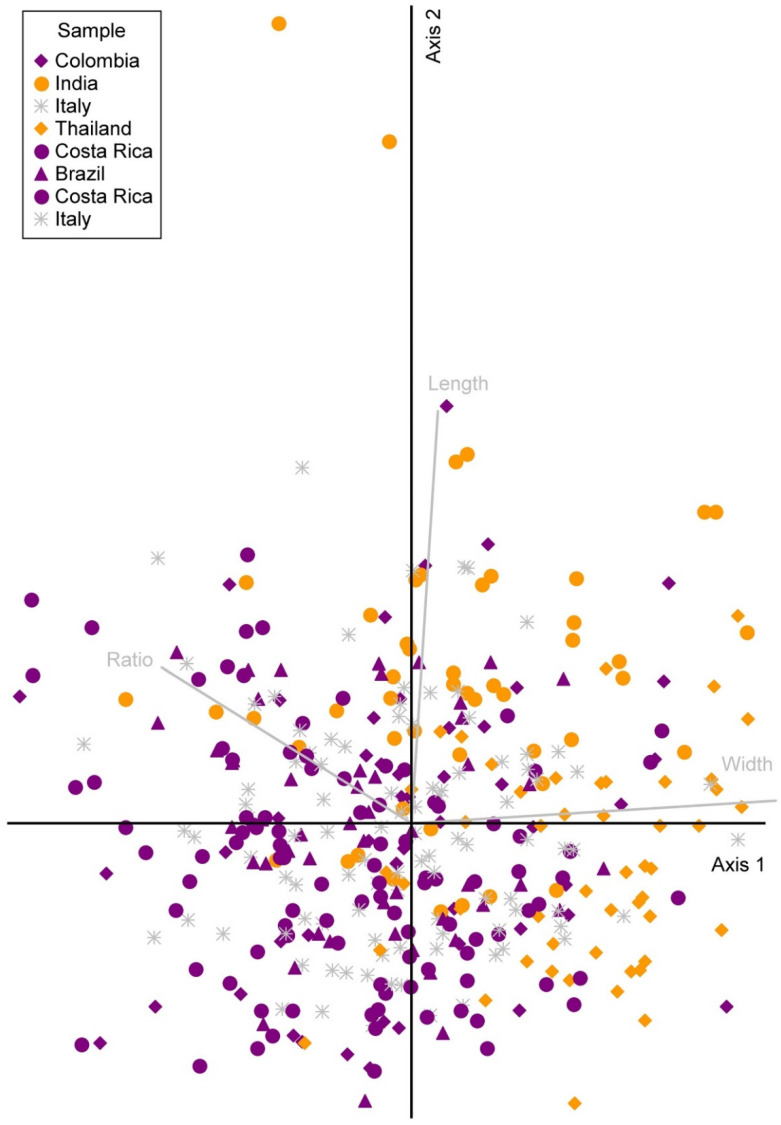
PCA plot of the ascospore length and width. Measurements were taken for eight specimens, which included fresh and herbarium specimens from 50 ascospores of each specimen. Brazil: Cáceres and Aptroot 11449; Colombia: Lindig 2865; Costa Rica: Tibell 8232, Tibell 8306; India: Kurz 1866; Italy: Beltramini s.n. (L-008798, L-996762); Thailand: MFLU 21-0135.

The PCA indicated a homogeneous, unimodal distribution of the ascospore size measurements, with only one large cluster, although the data on the ascospore width included two outliers (Figure 2). There was some tendency of the samples to differentiate according to region, especially regarding the ascospore width; the samples from the Neotropics clustered more towards the left and those from the Paleotropics more towards the right of the first axis, which largely corresponded to the ascospore width. Mean values for the ascospore length were 6.30 µm (Neotropics), 6.42 µm (Europe) and 6.77 µm (Paleotropics). The mean values for the ascospore width were 3.45 µm (Neotropics), 3.52 µm (Europe) and 3.97 µm (Paleotropics). The mean values for the Q value (ratio) were 1.84 (Neotropics), 1.81 µm (Europe) and 1.75 µm (Paleotropics).

This tendency was significant in terms of the ascospore width and length (ascospore length ANOVA: *f*-ratio = 13.8751, *p* < 0.00001; ascospore width ANOVA: *f*-ratio = 51.2113, *p* < 0.00001) but not for the Q value or the length:width ratio (ANOVA: *f*-ratio = 2.6403, *p* = 0.0726). The length and width differed significantly between regions 1 and 2 (Neotropics, Europe), on one hand, and region 3 (Paleotropics), on the other, but not between regions 1 and 2 (length according to post hoc Tukey HSD: 1 vs. 2: Q = 1.77, *p* = 0.4230; 1 vs. 3: Q = 7.04, *p* = 0.0000; 2 vs. 3: Q = 5.27 (*p* = 0.0007; width according to post hoc Tukey HSD: 1 vs. 2: Q = 2.18, *p* = 0.2736; 1 vs. 3: Q = 13.36, *p* = 0.0000; 2 vs. 3: Q = 11.19, *p* = 0.0000).

## 4. Taxonomy

### 4.1. Sphinctrinaceae M. Choisy, Bull. Mens. Soc. Linn. Soc. Bot. Lyon 19: 65 (1950)

Type genus: *Sphinctrina* Fr.

Syn.: *Mycocaliciaceae* A.F.W. Schmidt, Mitt. Staatsinst. Allg. Bot. Hamburg 13: 127 (1970).

Type genus: *Mycocalicium* Vain.

Notes: With the inclusion of *Mycocaliciaceae*, *Sphinctrinaceae* comprises seven genera, viz., *Brunneocarpos, Chaenothecopsis, Mycocalicium, Phaeocalicium, Pyrgidium, Sphinctrina* and *Stenocybe* [23]. Apparently, this is the only “discomycetous” family in *Eurotiomycetes* [66]. Several taxa in this family also produce coelomycetous and/or hyphomycetous anamorphs [25,27,29].

### 4.2. Pyrgidium Nyl., Flora, Regensburg 50: 3 (1867)

Index Fungorum number: IF 4617; faces of fungi number: FoF 12620.

Type species: *Pyrgidium montellicum* (Beltr.) Tibell, lichenologist 14(3): 239 (1982).

Notes: *Pyrgidium* was previously assigned to *Sphinctrinaceae*, without molecular data, and the present molecular study supports this placement. According to Tibell [5], the genus comprises a single species, *P. montellicum*, found mainly in the neotropics but also known from other tropical regions. *Pyrgidium* is characterized by its sessile to stalked ascomata, blackish brown and sclerotized exciple, either simple or 1-septate, and broadly ellipsoid to oval, dark brown, ornamented ascospores [5].

### 4.3. Pyrgidium montellicum (Beltr.) Tibell, Lichenologist 14(3): 239 (1982) (Figure 3 and Figure 4)

Index Fungorum number: IF 110065; faces of fungi number: FoF 10274.

Basionym: *Acolium montellicum* Beltr. 1858 [37].

Index Fungorum number: IF 110065; type: Italy. Bazzano, Beltramini s.n., ex Hb. Massalongo (UPS-Syntype).

Synonyms: 

*Pyrgidium leptoconium* (Nyl.) Nádv., Stud. Bot. Čechoslov. 5: 125 (1942).

Index Fungorum number: IF 369854.

**Figure 5:**
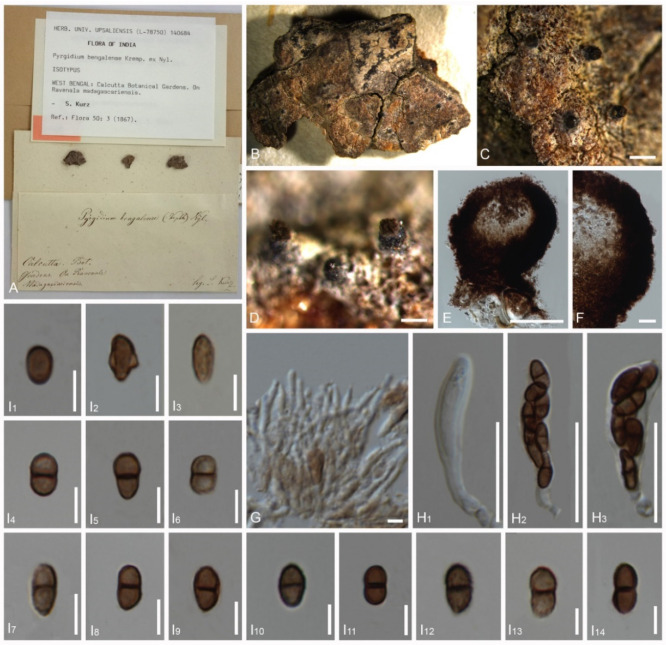
*Pyrgidium montellicum* (type material of *P. bengaliense*) (Kurz 1866, Isotype) (**A**). Details of the herbarium specimen, (**B**–**D**). Ascomata on substrate, (**E**). Vertical section through the ascoma, (**F**). Vertical section through the exciple, (**G**). Paraphyses, (**H_1_**–**H_3_**_)_. Asci, (**I_1_**–**I_4_**). Ascospores (in water), (**I_5_**–**I_14_**). Ascospores (in 5% KOH). Scale bars: (**C**) = 500 μm, (**D**) = 200 μm, (**E**) = 100 μm, (**F**) = 20 μm, (**G**) = 5 μm, (**H_1_**–**H_3_**) = 20 μm, (**I_1_**–**I_14_**) = 5 μm.

*Pyrgidium bengaliense* Nyl., Flora, Regensburg 50: 3 (1867) (Tibell 1982) (Figure 5)

Index Fungorum number: IF 403559. Type: India, Calcutta Botanic Gardens, on bark of *Ravenala madagascariensis,* Kurz 66 (Kurz 1866, UPS-Isotype).

**Figure 6:**
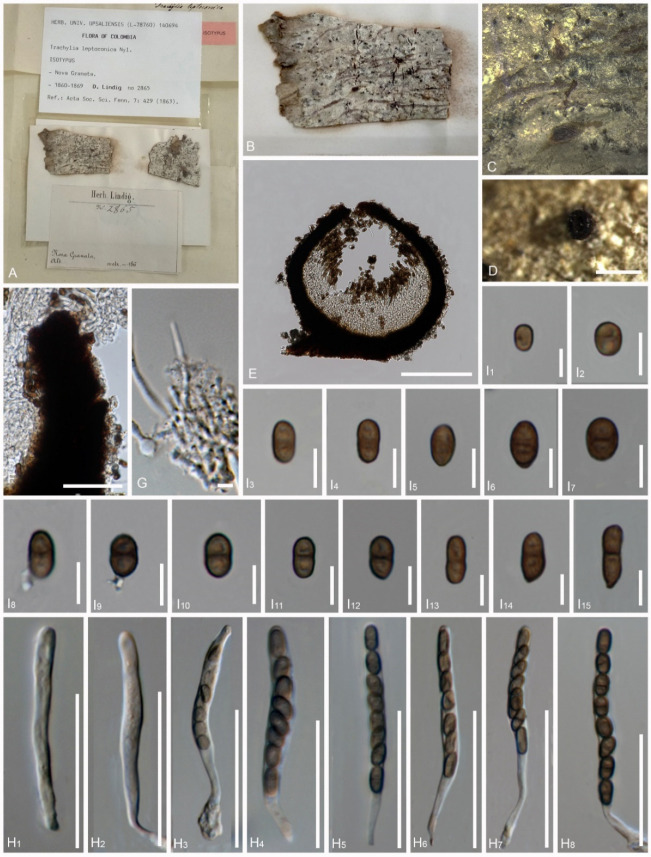
*Pyrgidium montellicum* (type material of *Trachylia leptoconia*) (Lindig 2865, isotype) (**A**). Details of the herbarium specimen, (**B**–**D**). Ascomata on substrate, (**E**). Vertical section through the ascoma, (**F**). Vertical section through the exciple, (**G**). Paraphyses, (**H_1_**–**H_6_**). Asci, (**I_1_**–**I_13_**,**I_15_**). Ascospores (in water), (**I_14_**). Ascospore (in 5% KOH). Scale bars: (**D**) = 500 μm, (**E**) = 100 μm, (**F**) = 20 μm, (**G**) = 5 μm, (**H_1_**–**H_6_**) = 30 μm, (**I_1_**–**I_15_**) = 5 μm.

*Trachylia leptoconia* Nyl., Acta Soc. Sci. fenn. 7(2): 429 (1863) [37]. (Figure 6)

Index Fungorum Number: IF 407801; type: Colombia (Colombia), Nova Granata, Fusagasuga, Lindig. A (Lindig 2865, UPS-Isotype). 

*Saprobic* on bark. *Thallus* crustose, farinose or absent, and whitish. *Prothallus* is absent. *Photobiont* with loosely associated algae, sparsely present in the thallus, and mostly trentepohlioid or absent. Sexual morph: *Ascomata* are scattered, apothecial, rarely urn-shaped, 180–330 μm diam., 130–290 μm high (M = 255 × 210 μm), sessile to shortly stalked, almost sphaerical, mazaedioid, with a black disc. *Excipulum* of 10–55 μm thickened laterally, 27–90 μm thickened basally, mostly thickened basally and gradually becoming thinner towards the upper part, sometimes comprising sclerotized hyphae. *Mazaedium* filling the cavity of the ascoma and more or less projecting beyond the excipular edge. *Paraphyses* are 0.4–2 μm thick, septate, and unbranched. *Asci* of 18–56 × 3–9 μm (M = 37 × 6 μm, n = 30), cylindrical, 8-spored, unitunicate, tip-blunted, not narrowing towards the apex, with the ascus apex thickened in immaturity and reduced or inconspicuous in maturity, and short pedicellate. *Ascospores* of 4–10 × 2–5 μm (M = 7 × 3.5 μm, n = 40), broadly ellipsoid to oval, uniseriate to biseriate, and light to dark brown, (0-)1-septate, with a dark brown septum, small appendage present at one end in few ascospores, and with guttulates when immature, wall verrucose or with irregular, longitudinally arranged ridges. Asexual morph: unknown.

Notes: *Pyrgidium montellicum* has thus far been reported in Central and South America (Costa Rica, Colombia, Ecuador, Brazil, Argentina), Eurasia (Italy, Iran, Russia, China), the eastern Paleotropics (India, Sri Lanka, Thailand, Papua New Guinea) and Australasia [5,64,67,68,69,70,71] (https://www.gbif.org/species/3269679, accessed on 15 January 2022). The new collections of *Pyrgidium montellicum* studied here also confirm the previously reported presence of this taxon in Brazil, where it is known to be present in the Amazon [72], and the subtropical coast of Rio de Janeiro [5] and Thailand [69], which is now, for the first time, supported by molecular data. The Brazilian specimens were mostly over mature and the asci were difficult to observe.

The collection of *Pyrgidium montellicum* from Thailand and Brazil clustered together in the phylogenetic analysis, providing strong support for the accurate placement of this lineage. No significant morphological differences were observed between the specimens representing the fresh collections and the types known by three names currently synonymized under the name *P.* *montellicum*, excepting the tendency of the paleotropical collections from India and Thailand to produce larger and especially broader ascospores. Base pair comparisons between the Brazilian and the Thai specimens revealed about a 4.7% difference in the ITS markers, correlating with the differences in the ascospore size. Even without evident morphological differences, such sequence divergences have been used to distinguish morphologically cryptic species in other cases [73,74]. Indeed, several studies have been conducted to assess the potentially cryptic nature of mycocalicioid species due to the limited number of taxonomically useful characteristics [75,76]. However, differences in a single molecular marker may not be seen as sufficient for establishing species boundaries in a cryptic lineage, especially if few specimens have been sequenced [74]. Here, a case could be made for the separation of the paleotropical populations into different species, but since only a few specimens have been examined molecularly and morphologically, for the time being, we agree with Tibell [5] that only a single sub-cosmopolitan species should be presently recognized. If the analysis of more material supports the encountered differences, *P. bengaliensis* could be resurrected for the paleotropical material. Apart from the size of the ascospores, several other characteristics, such as color (brown to dark brown), septation (0-1-septate), ornamentation (verrucose, gattulates in immaturity and irregular ridges, longitudinally arranged), a thicker outer wall, and highly pigmented septa with constrictions at the septum, do not seem to provide any taxonomic value in this case (Table 3).

Material examined: Thailand, Chiang Mai, 128 Moo3, Bahn Pa Dheng, T. Pa Pae, A. Mae Taeng, on the bark of an unidentified tree, 10 September 2020, Vinodhini Thiyagaraja (MFLU 21-0135); Brazil, Rondônia, Porto Velho, Parque Circuito, on the bark of *Hevea brasiliensis*, 11 March 2012, André Aptroot and Marcela Eugenia da Silva Cáceres (Cáceres and Aptroot ISE 11449).

**Figure 7:**
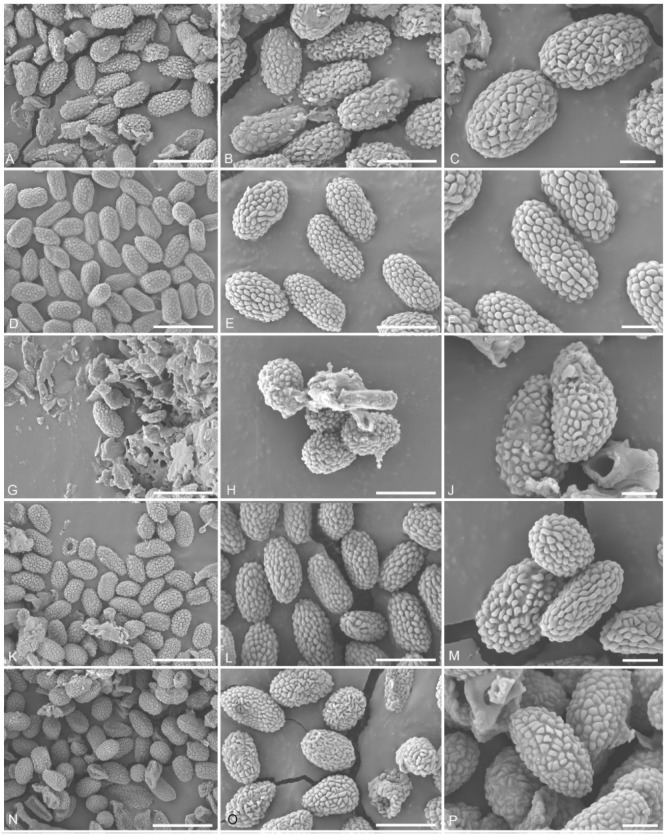
Ascospores of *Pyrgidium* species under SEM. (**A**–**C**). Kurz 1866, (**D**–**F**). Lindig 2865, (**G**–**J**). MFLU 21-0135, (**K**). L-996762, (**L**,**M**). Tibell 8306, (**N**). Tibell 8232, (**O**). L-008798, (**P**). L-996762. Scale bars: (**A**,**D**,**G**,**K**,**N**) = 10 μm, (**B**,**E**,**H**,**L**,**O**) = 5 μm, (**C**,**F**,**J**,**M**,**P**) = 2 μm.

Description of the fresh material of *Pyrgidium montellicum*: *Saprobic* on bark. *Thallus* crustose, whitish and endoperidermal. *Prothallus* absent. *Photobiont* absent or loosely associated algal cells sparsely present in the thallus, and trentepohlioid. **Sexual morph:** *Ascomata* scattered, apothecial, 22–330 μm diam., 130–195 μm high M = 277 × 162 μm), sessile to shortly stalked, almost sphaerical, mazaedioid, with a black disc. *Excipulum* 25–55 μm thick laterally, 30–60 μm thick basally, brown to black, prosoplectenchymatous, with the edge comprised of sclerotized hyphae, the edge of the excipulum turned inward in the topmost part in immature ascomata and eventually turned outward in the topmost part in maturity, laterally and gradually becoming thinner. *Mazaedium* filling the cavity of the ascoma and more or less projecting beyond the excipular edge. *Paraphyses* of 1–2 μm thick, septate and unbranched. *Asci* of 25–40 × 4–7 μm (M = 32 × 5.5 μm, n = 30), cylindrical, shortly pedicellate, 8-spored, unitunicate, tip-blunted and not narrowing towards the apex, with the ascus apex thickened when immature and reduced or inconspicuous when mature. *Ascospores* of 5–9 × 2.5–4.5 μm (M = 5.5 × 3.5 μm, n = 40), broadly ellipsoid to oval, uniseriate to biseriate, light to dark brown and (0-)1-septate, with a dark brown septum, small appendage present at one end in a few ascospores, with guttulates when immature, wall verrucose or with irregular, longitudinally arranged ridges (Figure 7K–P). Asexual morph: unknown. 

The types of the three synonyms of *Pyrgidium montellicum* are characterized as follows:

Description of the type *Acolium* *montellicum*: *Saprobic* on bark. *Thallus* crustose, farinose and whitish. *Prothallus* absent. *Photobiont* with loosely associated algae, sparsely present in thallus, mostly trentepohlioid or absent. Sexual morph: *Ascomata* scattered, apothecial, rarely urn-shaped, 180–225 μm diam., 160–290 μm high (M = 202 × 225 μm), sessile to shortly stalked, almost sphaerical, mazaedioid, with a black disc. *Excipulum* 10–40 μm thickened laterally, 27–46 μm thickened basally, mostly thickened basally and gradually becoming thinner towards the upper part, sometimes comprising sclerotized hyphae. *Mazaedium* filling the cavity of the ascoma and more or less projecting beyond the excipular edge. *Paraphyses* 0.5–2 μm thick, septate and unbranched. *Asci* 25–40 × 2–6 μm (M = 32 × 4 μm, n = 30), cylindrical, 8-spored, unitunicate, tip-blunted and not narrowing towards the apex, with the ascus apex thickened in immaturity and reduced or inconspicuous in maturity, and short pedicellate. *Ascospores* 4–7 × 2–4 μm (M = 5.5 × 3 μm, n = 40), broadly ellipsoid to oval, uniseriate to biseriate, light to dark brown, (0-)1-septate, with a dark brown septum, small appendage present at one end in few ascospores, with guttulates when immature, wall verrucose or with irregular, longitudinally arranged ridges. Asexual morph: unknown.

Description of the type *Pyrgidium* *bengaliense*: *Saprobic* on bark. *Thallus* inconspicuous, pruinose around the ascomata. *Prothallus* absent. *Photobiont* absent. Sexual morph: *Ascomata* apothecial, 190–205 μm diam., 180–220 μm high (M = 197 × 200 μm, n = 10), not stalked, mazaedioid, with a black disc, sessile, scattered and almost spherical. *Excipulum* 30–55 μm thickened laterally, 60–90 μm thickened basally, brown to black, prosoplectenchymatous and comprising some sclerotized hyphae. *Mazaedium* filling the cavity of the fruit body and more or less projecting beyond the excipular edge. *Paraphyses* 1.2–1.8 μm thick, septate and simple. *Asci* 18–33 × 4–9 μm (M = 25.5 × 6.5 μm, n = 30), cylindrical, 8-spored, unitunicate, tip-blunted and not narrowing towards the apex, with the ascus apex thickened in immaturity and reduced or inconspicuous in maturity, and short pedicellate. *Ascospores* 5.5–10 × 3–5 μm (M = 7.75 × 4 μm, n = 40, ellipsoidal, overlapping bi-seriate, light brown to brown, 0-1-septate, with a slightly dark brown septum, sometimes constricted at the septum, with gattulates when immature, verrucose, irregular ridges that are longitudinally arranged (Figure 7G–J). Asexual morph: undetermined. 

Description of the type *Trachylia leptoconia*: *Saprobic* on bark. *Thallus* crustose and farinose. *Prothallus* absent. *Photobiont* absent. Sexual morph: *Ascomata* apothecial, 220–240 μm diam., 220–235 μm high (M = 230 × 227 μm, n = 10), not stalked, mazaedioid, with a black disc, sessile, scattered and almost spherical. *Mazaedium* filling the cavity of the fruit body and more or less projecting beyond the excipular edge. *Excipulum* 22–28 μm thickened laterally, 35–55 μm thickened basally, brown to black, prosoplectenchymatous, hardly compromise any sclerotized hyphae, thickened basally and gradually becoming thinner towards the upper part. *Paraphyses* 1.2–2 μm thick, septate and simple. *Asci* 34–56 × 4–5 μm (M = 45 × 4.5 μm, n = 30), cylindrical, 8-spored, unitunicate and tip-blunted, with the ascus apex thickened when immature and reduced or inconspicuous when mature, and short pedicellate. *Ascospores* 4.5–9 × 2–5 μm (M = 6.75 × 3.5 μm, n = 40), ellipsoidal, uniseriate, overlapping, light brown to brown, 0-1-septate, with a dark brown septum, small appendage present at one end in few ascospores, with gattulates when immature, and verrucose, irregular ridges that are longitudinally arranged (Figure 7D–F). Asexual morph: undetermined. 

## 5. Discussion

Although *Pyrgidium montellicum* is usually considered a saprotrophic taxon, several studies reported *Trebouxia* or allied cystococcaceous alga as photobionts, and some defined the taxon as commensal on lecanoralean lichens [6,37]. In addition, *P.* *montellicum* may serve as a host for lichenicolous fungi, such as *Chaenothecopsis rubina* Tibell [37]. In the material assessed in the present study, including both the fresh and historical specimens, no obligate and stable association with a particular photobiont was observed, but some specimens showed a weak association with trentepohlioid algae. Nádvorník [36] reported the presence of a *Trentepohlia* photobiont in the original material of *Pyrgidium leptoconium,* but this was not seen in the isotype material examined here. These findings support the notion that algal associations are accidental or facultative in the case of *P. montellicum* and that the taxon is primarily saprotrophic [37]. A similar situation can be observed with other borderline lichenized fungi, such as *Arthopyrenia salicis* A. Massal., *Cresporhaphis macrospora* (Eitner) M.B. Aguirre, *Requienella seminuda* (Pers.) Boise and *Splanchnonema lichenisatum* Aptroot and K.H. Moon, which are facultatively associated with various photobionts but were also recorded without any algal associations [77,78,79]. Notably, these lineages are found in predominantly non-lichenized clades, such as *Dothideomycetes* and *Sordariomycetes* [77,78,79,80], suggesting initial evolutionary attempts at lichenization in these lineages. On the other hand, several bark-inhabiting fungi are known to have emerged from largely lichenized clades, and their saprotrophic mode evolved secondarily from their lichenized ancestors [81,82,83].

*Eurotiomycetes* comprise lichenized and lichenicolous lineages mainly in the orders of *Pyrenulales* and *Verrucariales* within the subclass of *Chaetothyriomycetidae* [14]. Among these, *Pyrenula coryli* A. Massal. has been recorded as non-lichenized [84]. *Verrucariales* largely encompass lichenized species or mycophycobioses [85,86]. In contrast, saprotrophic species are mainly found in *Mycocaliciales* within the subclass *Mycocaliciomycetidae*, in the genera *Brunneocarpos*, *Chaenothecopsis*, *Mycocalicium*, *Phaeocalicium*, *Stenocybe* and *Strongyleuma.* *Chaenothecopsis* also comprise lichenicolous taxa, and one species, *C. pusilla* (Ach.) A.F.W. Schmidt, was found to be facultatively associated with algae [87,88]. 

Ascospore characteristics, such as color, size, septation and ornamentation, have been used for the generic and species delineation of mycocalicioid fungi [5,37]. Ascospores have been mostly recorded as 1-septate in *Pyrgidium* [5,37], although aseptate ascospores were also frequently observed in the material examined in this study. Sometimes, appendages were observed at one end of the ascospores (Tibell 8306; Tibell 8232; Lindig 2865; MFLU 21-0135;). The ascospores of *Pyrgidium* have small warts or ridges that were visible under the scanning electron microscope. Nylander [35] defined the ascospore dimensions as 5–9 × 3–4 μm for *Pyrgidium bengaliense*, and the re-examination of the isotype material resulted in dimensions of 5.5–10 × 3–5 μm. The ascospore dimensions of the original material of *P. leptoconia* were given as 6–8 × 4–4.5 μm, whereas the isotype revealed measurements of 4.5–9 × 2–5 μm. *Pyrgidium montellicum* was originally described as having 5.5–7 × 3–4.5 μm large ascospores, while our measurements were 4–7 × 2–4 μm for the four non-type specimens. Variations in then ascospore size revealed limited clustering tendencies according to the geographic region, especially regarding the width, and both the length and width showed minor but significant differences between the groups from the Neotropics and Europe, on one hand, and the Paleotropics, on the other. However, due to the limited material examined, we refrained from dividing *P. montellicum* into more than one species at this point in time. 

Tibell [37] described *P. montellicum* as having perithecial, urn-shaped ascomata, but later studies described them as apothecial [5,88]. The re-examination of several collections revealed both urn-shaped perithecial and apothecial ascomata, suggesting that these morphologies intergrade during ontogeny. The asci of all mycocalicioid fungi are known to arise from croziers [89,90], and careful microscopic observations revealed the presence of croziers in all the studied specimens of *Pyrgidium*. The ascus apex was almost reduced in maturity and could only be observed in the immature stage or after adding 5% KOH. Overall, considering all these characteristics, there are no clear-cut differences between the examined specimens, which, at present, supports the argument of Tibell [5] that we should consider *Pygridium* as a monospecific genus. However, the variation in the ITS associated with geography (Brazil vs. Thailand) warrants further attention and should be investigated using more material so as to assess the potential cryptic speciation through vicariance.

## Figures and Tables

**Figure 3:**
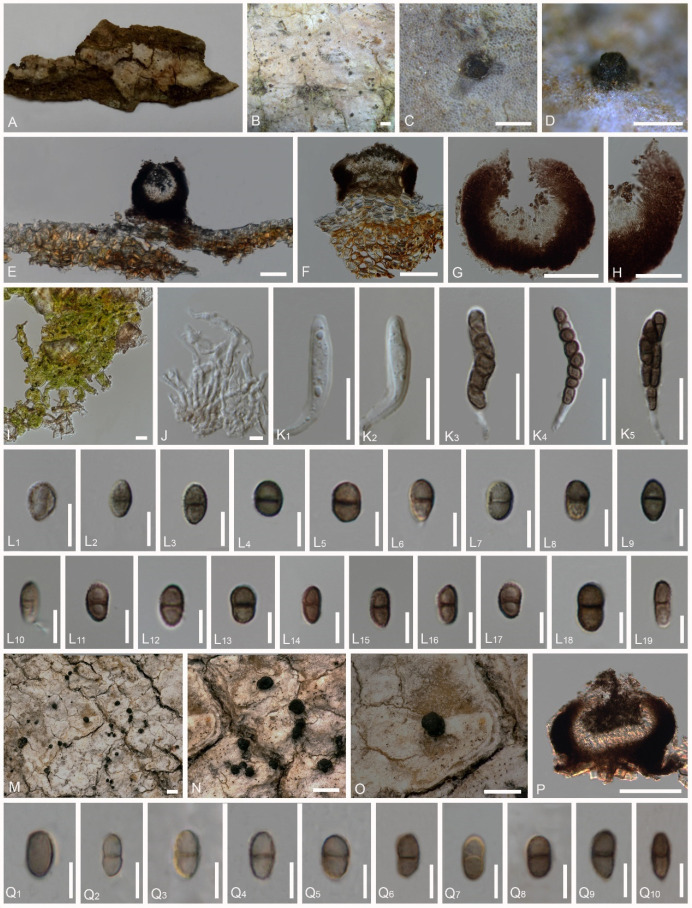
*Pyrgidium montellicum* (Thailand: MFLU 21-0135, Brazil: Cáceres and Aptroot 11449), (**A**–**D**). Ascomata on substrate (MFLU 21-0135), (**E**–**G**). Vertical section through the ascoma (MFLU 21-0135), (**H**). Vertical section through the exciple (MFLU 21-0135), (**I**). Loosely associated algae (MFLU 21-0135), (**J**). Paraphyses (MFLU 21-0135), (**K_1_**–**K_5_**). Asci (MFLU 21-0135), (**L_1_**–**L_10_**). Ascospores in water (MFLU 21-0135), (**L_11_**–**L_19_**). Ascospores in 5% KOH (MFLU 21-0135), (**M**–**O**). Ascomata on substrate (Cáceres & Aptroot 11449), (**P**). Vertical section through the ascoma (Cáceres and Aptroot 11449). (**Q_1_**–**Q_10_**_)_. Ascospores in water (Cáceres and Aptroot 11449). Scale bars: (**B**–**D**), (**M**–**O**) = 500 μm, (**C**,**D**) = 200 μm, (**E**–**G**), (**P**) = 100 μm, (**H**) = 50 μm, (**I**) = 10 μm, (**J**) = 5 μm, (**K_1_**–**K_5_**) = 20 μm, (**L_1_**) = 10 μm, (**L_2_**–**L_19_**), (**Q_1_**–**Q_10_**) = 5 μm.

**Figure 4:**
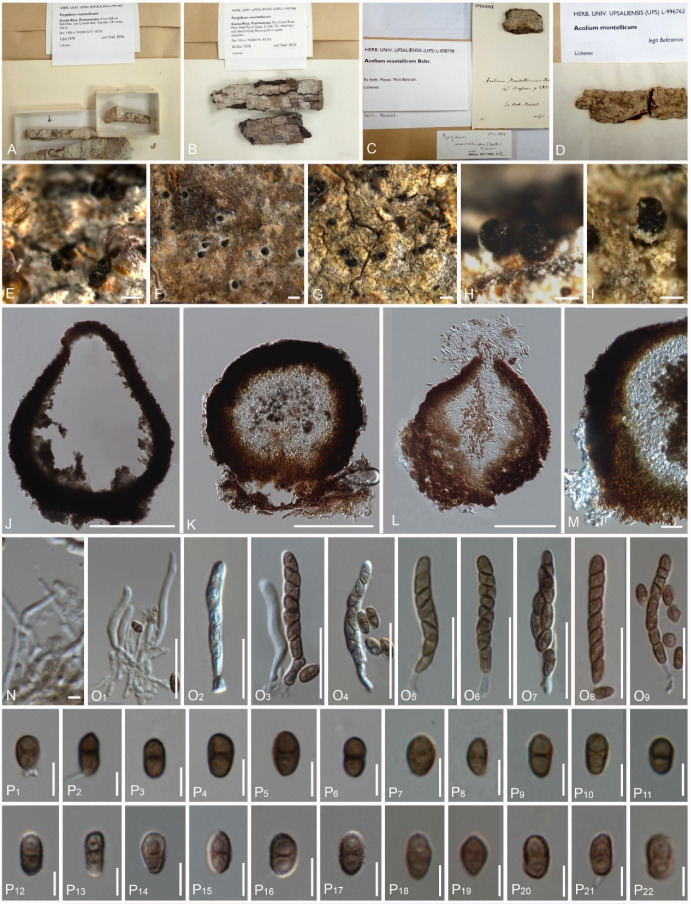
*Pyrgidium montellicum* (type materials of *Acolium* *montellicum* (L-008798, L-996762) and non-type materials of *P. montellicum* (Tibell 8306, Tibell 8232)), (**A**–**D**). Details of herbarium specimens (**A**). Tibell 8306, (**B**). Tibell 8232, (**C**). L-008798, (**D**). L-996762, (**E**–**I**). Ascomata on substrate (**E**,**H**). Tibell 8306, (**F**). Tibell 8232, (**G**,**I**). L-008798, (**J**–**L**). Vertical section through the exciple, (**J**). Tibell 8306, (**K**). Tibell 8232, (**L**). L-996762, (**M**). Tibell 8232, (**N**). L-996762, (**O_1_**–**O_9_**). Asci, (**O_1_**–**O_4_**,**O_7_**). L-996762, (**O_5_**,**O_6_**). L-008798, (**O_8_**,**O_9_**). Tibell 8232. Ascospores ((**P_1_**–**P_22_**), (**P_1_**–**P_6_**). Tibell 8306, (**P_7_**–**P_8_**). L-008798, (**P_12_**–**P_16_**). 1981 L-996762, (**P_17_**–**P_22_**). Tibell 8232)). Scale bars: (**E**,**F**) = 1000 μm, (**G**,**H**) = 500 μm, (**J**–**L**) = 100 μm, (**M**) = 20 μm, (**K**) = 5 μm, (**O_1_**–**O_9_**) = 20 μm, (**P_1_**–**P_22_**) = 5 μm.

**Table 1:** Gene regions, respective primer pairs and PCR conditions used in the study.

Gene Region	Primers	PCR Condition	References
ITS	ITS4 and ITS5	95 °C: 4 min, (94 °C: 1 min, 54 °C: 1 min, 72 °C: 45 s) × 35 cycles 72 °C: 5 min	[7,42]
LSU	LROR and LR5	94 °C: 5 min, (94 °C: 40 s, 52 °C: 40 s, 72 °C: 40 s) × 35 cycles 72 °C: 10 min	[43,44]
SSU	NS1 and NS4	95 °C: 15 min, (95 °C: 27 s, 54–56 °C: 30 s, 72 °C: 1 min) × 35 cycles 72 °C: 5 min	[42,45]
mtSSU	mtSSU1 and mtSSU3R	94 °C: 3 min, (94 °C: 3 min, 52 °C: 1 min, 72 °C: 1 min) × 35 cycles 72 °C: 10 min	[46]

**Table 2:** Taxa names, strain numbers and corresponding GenBank accession numbers of the LSU and ITS sequences used in the phylogenetic analyses. The newly generated sequences are shown in bold face.

Taxa	Strain	GenBank Accessions		
		LSU	ITS	References
*Brunneocarpos banksiae*	CBS 141465	NG_066277	-	[29]
*Chaenothecopsis consociata*	Tibell 22472	DQ008999	AY795851	[48]
*Chaenothecopsis khayensis*	H:JR 04G058	-	NR_120165	[57]
*Chaenothecopsis resinophila*	H:JR 000424	JX122782	JX122780	[58]
*Chaenothecopsis schefflerae*	Rikkinen 13183	KY499967	KY499965	[59]
*Chaenothecopsis subparoica*	Tretiach (hb. Tretiach)	-	AY795869	[48]
*Chaenothecopsis viridireagens*	H:Tuovila 09-068	JX119117	JX119108	[58]
*Chaenothecopsis pallida*	H:JR 010652	JX122781	JX122779	[58]
*Chaenothecopsis pusiola*	Tibell 15884 (UPS)	-	AY795865	[48]
*Chaenothecopsis fennica*	Tibell 16024 (UPS)	AY795995	AY795857	[48]
*Chaenothecopsis sitchensis*	Tuovila 06-33 (TUR)	KF157988	-	[59]
*Chaenothecopsis golubkovae*	Titov 6707 (UPS)	AY795996	AY795859	[48]
*Chaenothecopsis viridireagens*	Tibell 22803 (UPS)	DQ013257	AY795872	[48]
*Fusichalara minuta*	CBS 709.88	KX537758	KX537754	[60]
*Mycocalicium subtile*	Tibell 16744 (UPS)	AY796004	-	[48]
*Mycocalicium subtile*	Tibell 17164 (UPS)	AY796005	-	[48]
*Mycocalicium albonigrum*	Tibell 19038	AY796001	AF223966	[48]
*Phaeocalicium curtisii*	BIOUG24047-F02	-	KT695401	[61]
*Phaeocalicium populneum*	Tibell 19286 (UPS)	AY796009	AY795874	[48]
*Phaeocalicium praecedens*	Tuovila 09-240 (TUR)	KC590486	KC590481	[27]
*Pyrenula minutispora*	ABL AA11877	-	KT820119	[62]
*Pyrenula nitida*	F 5929	DQ329023	JQ927458	[63,64]
***Pyrgidium montellicum***	**MFLU 21-0135a**	**ON979678**	**ON979674**	**This study**
***Pyrgidium montellicum***	**MFLU 21-0135b**	**-**	**OP094605**	**This study**
***Pyrgidium montellicum***	**Cáceres and Aptroot 11449**	**OP077215**	**ON979667**	**This study**
*Rhopalophora clavispora*	CBS 129.74	MH872573	KX537751	[60]
*Rhopalophora clavispora*	CBS 281.75	KX537756	KX537752	[50]
*Sphinctrina leucopoda*	Kalb 33829 (hb. Kalb)	AY796006	AY795875	[48]
*Sphinctrina turbinata*	AFTOL-ID 1721	EF413632	-	[14]
*Sphinctrina turbinata*	Tibell 22478 (UPS)	-	AY795876	[14]
*Stenocybe pullatula*	Tibell 17117 (UPS)	AY796008	AY795878	[48]
*Verrucaria inverecundula*	FILIC650-13	-	MK138796	[65]

**Table 3:** Morphological comparison of the herbarium and fresh specimens based on this study.

	*P.bengaliense* (Kurz 1866; Isotype UPS)	*Trachylia leptoconia* (Lindig 2865; Isotype UPS)	*Acolium montellicum* (L-008798; Syntype UPS)	*Acolium montellicum* (L-996762)	*P. montellicum* (Tibell 8306; Non-Type UPS)	*P. montellicm* Tibel (8232; Non-Type UPS)	*P. montellicum* (Cáceres and Aptroot 11449)	*P. montellicum* (MFLU 21-0135)
Thallus	Absent	Farinose	Farinose	Farinose	Farinose	Absent	Absent	Farinose
Ascomata width (μm)	190–205	220–240	215–225	185–190	180–195	190–205	225–235	180–330
Ascomata height (μm)	180–220	220–235	160–170	175–190	210–290	170–190	130–145	135–195
Exciple (lateral) (μm)	30–55	22–28	26–31	25–40	11–30	30–39	30–45	25–55
Exciple (base) (μm)	60–90	35–55	33–46	28–40	27–38	27–42	34–46	30–60
Paraphyses width (μm)	0.9–2	1–2	0.8–1.6	1–2	0.7–1.7	0.4–1.6	–	1–2
Asci length (μm)	18–33	34–56	31–36	29–39	28–32	25–40	–	25–40
Asci width (μm)	4–9	4–5	3.5–5	3–6	3–4	2–5	–	5–7
Ascospore length (μm)	5.5–10	4.5–9	4–5.5	5–7	4.5–7	4.5–7	5–8	6–9
Ascospore width (μm)	3–5	2–5	2.6–3.8	2–4	2.5–4	2–4	2.7–4	2.5–4.5
Q value	1.94	1.82	1.76	1.85	1.73	1.98	1.81	1.55
No. of septates per ascospore	0–1	0–1	0–1	0–1	0–1	0–1	0–1	0–1
Geographical occurrence	India	Colombia	Italy	Italy	Costa Rica	Costa Rica	Brazil	Thailand

## Data Availability

Not applicable.

## References

[1] Prieto M., Baloch E., Tehler A., Wedin M. (2013). Mazaedium evolution in the *Ascomycota* (Fungi) and the classification of mazaediate groups of formerly unclear relationship. Cladistics.

[2] Rikkinen J., Meinke S.K.L., Grabenhorst H., Gröhn C., Kobbert M., Wunderlich J., Schmidt A.R. (2018). Calicioid lichens and fungi in amber–Tracing extant lineages back to the Paleogene. Geobios.

[3] Fries E. (1817). Lichenum dianome nova. Lund.

[4] Wedin M., Tibell L. (1997). Phylogeny and evolution of *Caliciaceae*, *Mycocaliciaceae*, and *Sphinctrinaceae* (*Ascomycota*), with notes on the evolution of the prototunicate ascus. Canad. J. Bot..

[5] Tibell L. (1996). Caliciales—Flora Neotropica, Monograph 69.

[6] Tibell L., Wedin M. (2000). *Mycocaliciales*, a new order for nonlichenized calicioid fungi. Mycologia.

[7] Temu S.G., Tibell S., Tibuhwa D.D., Tibell L. (2019). Crustose Calicioid Lichens and Fungi in Mountain Cloud Forests of Tanzania. Microorganisms.

[8] Vainio E.A. (1927). Lichenographia fennica III*, Coniocarpeae*. Acta Soc. Pro Fauna Flora Fenn..

[9] Schmidt A. (1970). Anatomisch-taxonomische Untersu- chungen an Europaischen Arten der Flechtenfamilie *Caliciaceae*. Mitteilung zur Staatsinstitut fuXr Aligemeine Botanik, Hamburg. Hambg. Staatsinst. Allg. Bot. Mitt..

[10] Tibell L.B. (1994). Distribution patterns and dispersal strategies of *Caliciales*. Bot. J. Lin. Soc..

[11] Tibell L. (1996). *Phaeocalicium* (*Mycocaliciaceae*, *Ascomycetes*) in Northern Europe. Annales Botanici Fennici.

[12] Tibell L.B. (1984). A reappraisal of the taxonomy of *Caliciales*. Beih. Nova Hedwig..

[13] Prieto M., Wedin M. (2017). Phylogeny, taxonomy and diversification events in the *Caliciaceae*. Fung Divers..

[14] Geiser D.M., Gueidan C., Miadlikowska J., Lutzoni F., Kauff F., Hofstetter V., Fraker E., Schoch C.L., Tibell L., Untereiner W.A. (2006). *Eurotiomycetes*: *Eurotiomycetidae* and *Chaetothyriomycetidae*. Mycologia.

[15] Hibbett D.S., Binder M., Bischoff J.F., Blackwell M., Cannon P.F., Eriksson O.E., Huhndorf S., James T., Kirk P.M., Lücking R. (2007). A higher-level phylogenetic classification of the Fungi. Mycol. Res..

[16] Lumbsch H.T., Huhndorf S.M. (2007). Outline of *Ascomycota*–2007. Myconet.

[17] Schmitt I. (2011). 8 Fruiting Body Evolution in the *Ascomycota*: A Molecular perspective integrating lichenized and non-lichenized groups. Evolution of Fungi and Fung. Like Organisms.

[18] Sadowski E.M., Beimforde C., Gube M., Rikkinen J., Singh H., Seyfullah L.J., Heinrichs J., Nascimbene P.C., Reitner J., Schmidt A.R. (2012). The anamorphic genus *Monotosporella* (*Ascomycota*) from Eocene amber and from modern Agathis resin. Fung Biol..

[19] Gueidan C., Aptroot A., da Silva Cáceres M.E., Badali H., Stenroos S. (2014). A reappraisal of orders and families within the subclass *Chaetothyriomycetidae* (*Eurotiomycetes*, *Ascomycota*). Mycol. Prog..

[20] Geiser D.M., LoBuglio K.F., Gueidan C. (2015). 5 *Pezizomycotina*: *Eurotiomycetes*. Syst. Evol..

[21] Wijayawardene N.N., Hyde K.D., Rajeshkumar K.C., Hawksworth D.L., Madrid H., Kirk P.M., Braun U., Singh R.V., Crous P.W., Kukwa M. (2017). Notes for genera–*Ascomycota*. Fung Divers..

[22] Wijayawardene N.N., Hyde K.D., Divakar P.K., Rajeshkumar K.C., Weerahewa D., Delgado G., Wang Y., Fu L. (2018). Notes for genera update—*Ascomycota*: 6616-6821. Mycosphere.

[23] Wijayawardene N., Hyde K., Al-Ani L., Tedersoo L., Haelewaters D., Rajeshkumar K.C., Zhao R.-L., Aptroot A., Saxena R., Tokarev Y. (2020). Outline of Fungi and fungus-like taxa. Mycosphere.

[24] Wijayawardene N.N., Hyde K.D., Dai D.Q., Sánchez-García M., Goto B.T., Saxena R.K., Erdogdu M., Selçuk F., Rajeshkumar K.C., Aptroot A. (2022). Outline of Fungi and fungus-like taxa-2021. Mycosphere.

[25] Titov A., Tibell L. (1993). *Chaenothecopsis* in the Russian Far East. Nord. J. Bot..

[26] Tibell L., Titov A. (1995). Species of *Chaenothecopsis* and *Mycocalicium* (*Caliciales*) on exudate. Bryologist.

[27] Tuovila H., Davey M.L., Yan L., Huhtinen S., Rikkinen J. (2014). New resinicolous *Chaenothecopsis* species from China. Mycologia.

[28] Ariyawansa H.A., Hyde K.D., Jayasiri S.C., Buyck B., Chethana K.W.T., Dai D.Q., Dai Y.C., Daranagama D.A., Jayawardena R.S., Lücking R. (2015). Fungal diversity notes 111–252—Taxonomic and phylogenetic contributions to fungal taxa. Fung. Divers..

[29] Crous P.W., Wingfield M.J., Richardson D.M., Le Roux J.J., Strasberg D., Edwards J., Roets F., Hubka V., Taylor P.W.J., Heykoop M. (2016). Fungal Planet description sheets: 400–68. Persoonia.

[30] Choisy M. (1950). Catalogue des lichens de la region Lyonnaise. Bull. Mens. Soc. Linn. Lyon.

[31] Muniz D., Llop E., Hladun N.L. (2013). *Sphinctrina paramerae*, a new Mediterranean lichenicolous species with non-septate spores. Lichenologist.

[32] Jaklitsch W., Baral H.-O., Lücking R., Lumbsch H.T., Frey W. (2016). Syllabus of Plant Families–Adolf Engler’s Syllabus der Pflanzenfamilien.

[33] Lücking R., Hodkinson B.P., Leavitt S.D. (2017). Corrections and amendments to the 2016 classification of lichenized fungi in the *Ascomycota* and *Basidiomycota*. Bryologist.

[34] Falswal A., Bhandari B.S. (2021). A New Lichenicolous Fungus from Garhwal Himalayan Region of Uttarakhand, India. Acta Bot..

[35] Nylander W. (1867). Addenda nova ad Lichenographiam Europaeam. Continuatio quarta. Flora.

[36] Nádvorník J. (1942). Systematische Übersicht der mitteleuropäischen Arten der Flechtenfamilie *Caliciaceae*. Stud. Bot. Cech..

[37] Tibell L. (1982). *Caliciales* of Costa Rica. Lichenologist.

[38] (2022). Index Fungorum. http://www.indexfungorum.org/names/Names.asp.

[39] (2022). Mycobank. https://www.mycobank.org/page/Simple%20names%20search.

[40] Jayasiri S.C., Hyde K.D., Ariyawansa H.A., Bhat J., Buyck B., Cai L., Dai Y.C., Abd-Elsalam K.A., Ertz D., Hidayat I. (2015). The Faces of Fungi database: Fungal names linked with morphology, phylogeny and human impacts. Fung. Divers..

[41] Dissanayake A.J., Bhunjun C.S., Maharachchikumbura S.S.N., Liu J.K. (2020). Applied aspects of methods to infer phylogenetic relationships amongst fungi. Mycosphere.

[42] White T.J., Bruns T., Lee S.J.W.T., Taylor J.W. (1990). Amplification and direct sequencing of fungal ribosomal RNA genes for phylogenetics. PCR Protoc. Guide Methods Appl..

[43] Vilgalys R., Hester M. (1990). Rapid genetic identification and mapping of enzymatically amplified ribosomal DNA from several *Cryptococcus* species. J. Bacteriol..

[44] Messuti M.I., Vidal-Russell R., Amico G.C., Lorenzo L.E. (2012). *Chaenothecopsis quintralis*, a new species of calicioid fungus. Mycologia.

[45] Schoch C.L., Sung G.H., López-Giráldez F., Townsend J.P., Miadlikowska J., Hofstetter V., Robbertse B., Matheny P.B., Kauff F., Wang Z. (2009). The *Ascomycota* tree of life: A phylum-wide phylogeny clarifies the origin and evolution of fundamental reproductive and ecological traits. Syst. Boil..

[46] Zoller S., Scheidegger C., Sperisen C. (1999). PCR primers for the amplification of mitochondrial small subunit ribosomal DNA of lichen-forming ascomycetes. Lichenologist.

[47] Swindell S.R., Plasterer T.N. (1991). Seqman. Sequence Data Analysis Guidebook.

[48] Tibell L., Vinuesa M. (2005). *Chaenothecopsis* in a molecular phylogeny based on nuclear rDNA ITS and LSU sequences. Taxon.

[49] Katoh K., Rozewicki J., Yamada K.D. (2019). MAFFT online service: Multiple sequence alignment, interactive sequence choice and visualization. Brief. Bioinf..

[50] Hall T.A. (1999). BioEdit: A user–friendly biological sequence alignment editor and analysis program for Windows 95/98/NT. Nucl. Ac. Symp. Ser..

[51] Glez-Peña D., Gómez-Blanco D., Reboiro-Jato M., Fdez-Riverola F., Posada D. (2010). ALTER: Program-oriented conversion of DNA and protein alignments. Nucl. Acids Res..

[52] Stamatakis A. (2014). RAxML version 8: A tool for phylogenetic analysis and post–analysis of large phylogenies. Bioinformatics.

[53] Miller M.A., Pfeiffer W., Schwartz T. Creating the CIPRES Science Gateway for Inference of Large Phylogenetic Trees. Proceedings of the SC10 Workshop on Gateway Computing Environments (GCE10).

[54] Huelsenbeck J.P., Ronquist F. (2001). MRBAYES: Bayesian inference of phylogenetic trees. Bioinformation.

[55] Nylander J.A.A. (2004). MrModeltest 2.0. Program Distributed by the Author.

[56] Rambaut A. (2014). FigTree. Version 1.4. 2.

[57] Schoch C.L., Robbertse B., Robert V., Vu D., Cardinali G., Irinyi L., Meyer W., Nilsson R.H., Hughes K., Miller A.N. (2014). Finding needles in haystacks: Linking scientific names, reference specimens and molecular data for Fungi. Database.

[58] Tuovila H., Schmidt A.R., Beimforde C., Dörfelt H., Grabenhorst H., Rikkinen J. (2013). Stuck in time—A new *Chaenothecopsis* species with proliferating ascomata from Cunninghamia resin and its fossil ancestors in European amber. Fung. Divers..

[59] Beimforde C., Tuovila H., Schmidt A.R., Lee W.G., Gube M., Rikkinen J. (2017). *Chaenothecopsis schefflerae* (*Ascomycota*: *Mycocaliciales*): A widespread fungus on semi-hardened exudates of endemic New Zealand *Araliaceae*. N. Zeal. J. Bot..

[60] Réblová M., Untereiner W.A., Štěpánek V., Gams W. (2017). Disentangling *Phialophora* section *Catenulatae*: Disposition of taxa with pigmented conidiophores and recognition of a new subclass, *Sclerococcomycetidae* (*Eurotiomycetes*). Mycol. Progr..

[61] Telfer A.C., Young M.R., Quinn J., Perez K., Sobel C.N., Sones J.E., Levesque-Beaudin V., Derbyshire R., Fernandez-Triana J., Rougerie R. (2015). Biodiversity inventories in high gear: DNA barcoding facilitates a rapid biotic survey of a temperate nature reserve. Biodivers. Data J..

[62] Gueidan C., Aptroot A., da Silva Cáceres M.E., Binh N.Q. (2016). Molecular phylogeny of the tropical lichen family *Pyrenulaceae*: Contribution from dried herbarium specimens and FTA card samples. Mycol. Prog..

[63] Del Prado R., Schmitt I., Kautz S., Palice Z., Lücking R., Lumbsch H.T. (2006). Molecular data place *Trypetheliaceae* in *Dothideomycetes*. Mycol. Res..

[64] Weerakoon G., Wolseley P.A., Arachchige O., da Silva Cáceres M.E., Jayalal U., Aptroot A. (2016). Eight new lichen species and 88 new records from Sri Lanka. Bryologist.

[65] Pykälä J., Launis A., Myllys L. (2019). Taxonomy of the *Verrucaria kalenskyi-V. xyloxena* species complex in Finland. Nova Hedw..

[66] Ekanayaka A.H., Ariyawansa H.A., Hyde K.D., Jones E.B.G., Daranagama D.A., Phillips A.J.L., Hongsanan S., Jayasiri S.C., Zhao Q. (2017). *Discomycetes*: The apothecial representatives of the phylum *Ascomycota*. Fung. Divers..

[67] Titov A. (2001). Further notes on calicioid lichens and fungi from the Gongga Mountains (Sichuan, China). Lichenologist.

[68] Nayaka S., Upreti D.K. (2005). Status of lichen diversity in Western Ghats, India. Sahyadri E-News, Western Ghats BioDivers. Infor. Sys..

[69] Aptroot A., Saipunkaew W., Sipman H.J.M., Sparrius L.B., Wolseley P.A. (2007). New lichens from Thailand, mainly microlichens from Chiang Mai. Fung. Divers..

[70] Seaward M.R.D., Sipman H.J.M., Sohrabi M. (2008). A revised checklist of lichenized, lichenicolous and allied fungi for Iran. Sauteria.

[71] Nascimbene J., Marini L. (2010). Oak forest exploitation and black-locust invasion caused severe shifts in epiphytic lichen communities in Northern Italy. Sci. Tot. Env..

[72] da Silva Cáceres M.E., de Lima E.L., Nascimento A.A., Lücking R. (2014). *Liquens brasileiros*: Novas descobertas evidenciam a riqueza no Norte e Nordeste do país. Bol. Mus. Biol. Mello Leitão..

[73] Crespo A., Lumbsch H.T. (2010). Cryptic species in lichen-forming fungi. IMA Fungus.

[74] Sato H., Ohta R., Murakami N. (2020). Molecular prospecting for cryptic species of the *Hypholoma fasciculare* complex: Toward the effective and practical delimitation of cryptic macrofungal species. Sci. Rep..

[75] Vinuesa M.D.L.A., Sanches-Puelles J.M., Tibell L. (2001). Intraspecific variation in *Mycocalicium subtile* (*Mycocaliciaceae*) elucidated by morphology and the sequences of the ITS1-5.8 S-ITS2 region of rDNA. Mycol. Res..

[76] Nilsson R.H., Kristiansson E., Ryberg M., Hallenberg N., Larsson K.H. (2008). Intraspecific ITS variability in the kingdom Fungi as expressed in the international sequence databases and its implications for molecular species identification. Evol. Bioinfor..

[77] Ellis J.B., Everhart B.M. (1888). New species of fungi from various localities. J. Myc..

[78] Barr M.E. (1993). Redisposition of some taxa described by J. B. Ellis. Mycotaxon.

[79] Jaklitsch W.M., Gardiennet A., Voglmayr H. (2016). Resolution of morphology-based taxonomic delusions: *Acrocordiella, Basiseptospora, Blogiascospora, Clypeosphaeria, Hymenopleella, Lepteutypa*, *Pseudapiospora, Requienella, Seiridium* and *Strickeria*. Pers. Mol. Phyl. Evol. Fungi.

[80] Voglmayr H., Aguirre-Hudson M.B., Wagner H.G., Tello S., Jaklitsch W.M. (2019). Lichens or endophytes? The enigmatic genus *Leptosillia* in the *Leptosilliaceae* fam. nov. (*Xylariales*), and *Furfurella* gen. nov. (*Delonicicolaceae*). Pers. Mol. Phyl. Evol. Fungi.

[81] Hyde K.D., Hongsanan S., Jeewon R., Bhat D.J., McKenzie E.H.C., Jones E.B.G., Phookamsak R., Ariyawansa H.A., Boonmee S., Zhao Q. (2016). Fungal diversity notes 367–490: Taxonomic and phylogenetic contributions to fungal taxa. Fung. Divers..

[82] Thiyagaraja V., Lücking R., Ertz D., Wanasinghe D.N., Karunarathna S.C., Camporesi E., Hyde K.D. (2020). Evolution of non-lichenized, saprotrophic species of *Arthonia* (*Ascomycota*, *Arthoniales*) and resurrection of Naevia, with notes on Mycoporum. Fung. Divers..

[83] Thiyagaraja V., Lücking R., Ertz D., Karunarathna S.C., Wanasinghe D.N., Lumyong S., Hyde K.D. (2021). The evolution of life modes in *Stictidaceae*, with three novel taxa. J. Fung..

[84] Kossowska M., Faltynowicz W., Dimos-Zych M., Faltynowicz H., Patejuk K., Piegdon A., Buksakowska M., Jarema P. (2018). Additions to the lichen biota of the Sudety Mountains. I. Records from the Karkonosze Mountains. Acta Mycologica.

[85] Hawksworth D.L. (1988). The variety of fungal-algal symbioses, their evolutionary significance, and the nature of lichens. Bot. J. Lin. Soc..

[86] Kohlmeyer J., Volkmann-Kohlmeyer B. (1998). A new marine *Xylomyces* on *Rhizophora* from the Caribbean and Hawaii. Fung. Divers..

[87] Wirth V. (1995). Die Flechten Baden-Württembergs. Teil 1.

[88] Groner U. (2006). The genus *Chaenothecopsis* (*Mycocaliciaceae*) in Switzerland, and a key to the European species. Lichenol..

[89] Nimis P.L., Puntillo D. Keys to the Lichens of Italy-08) Calicioid Species (Both Lichenized and Non-Lichenized). https://italic.units.it/flora/index.php?procedure=ext_key_home&key_id=1619.

[90] Tuovila H., Huhtinen S. (2020). New methods for mycocalicioid fungi. Lichenologist.

